# Desmoplastic Fibroblastoma Invading the Humerus

**DOI:** 10.1155/2020/9780263

**Published:** 2020-05-23

**Authors:** Tomoya Matsunobu, Akira Maekawa, Suguru Fukushima, Mao Jotatsu, Kosuke Makihara, Masanori Hisaoka, Yukihide Iwamoto

**Affiliations:** ^1^Department of Orthopaedic Surgery, Kyushu Rosai Hospital, Japan; ^2^Department of Musculoskeletal Oncology, National Cancer Center Hospital, Japan; ^3^Department of Pathology, Kyushu Rosai Hospital, Japan; ^4^Department of Pathology and Oncology, School of Medicine, University of Occupational and Environmental Health, Japan

## Abstract

Desmoplastic fibroblastoma (DFB) is an uncommon, benign, soft tissue tumor. The tumor most often presents as a slowly growing, painless soft tissue mass and is usually small. There have been only a few reports of patients with DFB who presented with bone invasion. Herein, we report the case of a 66-year-old woman with DFB with bone invasion in her left axilla. A lump under the left axilla was detected and was associated with pain and limited range of motion (ROM) of the shoulder. Computed tomography showed a soft tissue mass with invasion of the adjacent left humerus. Magnetic resonance imaging revealed a lesion with low signal intensity on T1- and T2-weighted images, and weak internal enhancement on postcontrast T1-weighted images with fat suppression. Histologic evaluation of a preoperative needle biopsy revealed DFB with FOSL1 expression. The tumor was marginally excised. Postoperative outpatient follow-up demonstrated a significant improvement in pain and ROM of the shoulder and no recurrence after 1 year. Even though DFB with bone invasion can cause pronounced clinical symptoms with pain and limited ROM, we conclude that simple excision is an adequate treatment.

## 1. Introduction

Desmoplastic fibroblastoma (DFB), also known as collagenous fibroma, is a rare, benign, soft tissue tumor that was first described by Evans in 1995 [1]. It usually manifests as a well-circumscribed, round or oval, painless, and slow-growing mass [2]. The vast majority of DFBs are 4 cm or smaller at the time of excision [2]. In the present article, we report the case of a relatively large and symptomatic DFB under the axilla, with aggressive bone invasion.

## 2. Case Report

**Figure 1:**
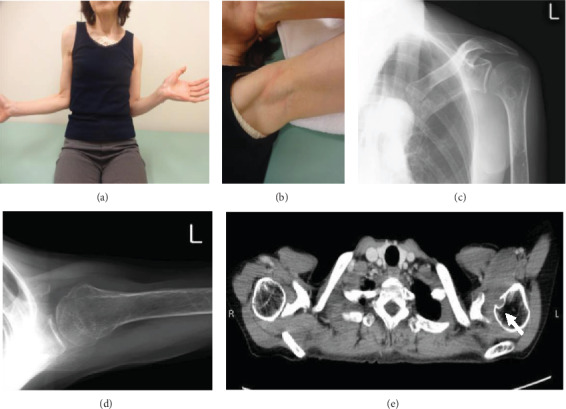
(a, b) Photograph of the patient at her first visit, with maximal external rotation of the shoulder (a), and photograph of the left axillary lump (b). Anteroposterior (c) and lateral (d) views on plain X-ray show a well-circumscribed, oval, osteolytic lesion with sclerotic margins in the lesser tubercle of the left humerus. (e) Contrast-enhanced computed tomography (CT) shows a soft tissue mass with invasion of the adjacent left humerus (arrow).

**Figure 2:**
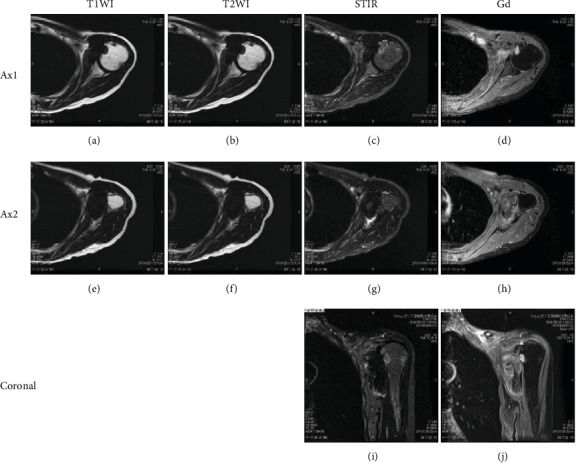
MRI images of the DFB in the left axilla with humeral invasion. (a–d) Raw axial images of the lesion with invasion of the underlying humerus. (e–h) Raw axial images of the lesion at its maximum diameter. (i–j) Raw coronal images of the lesion. First column (a, e), T1-weighted images. Second column (b, f), T2-weighted images. Third column (c, g, i), T2-weighted fat-suppression images. Fourth column (d, h, j), postcontrast T1-weighted images with fat suppression. The abbreviations: T1WI: T1-weighted images; T2WI: T2-weighted images; STIR: short TI inversion recovery; Gd: gadolinium-enhanced fat-suppressed T1-weighted images; Ax: axial images.

**Figure 3:**
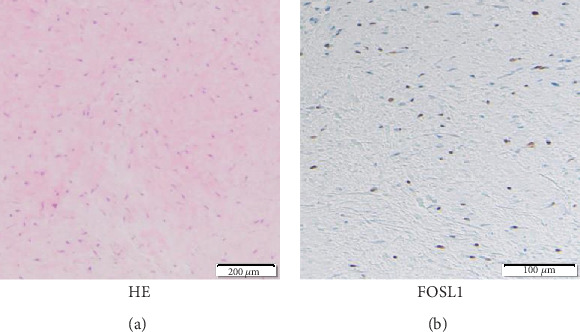
(a) Microscopic image (hematoxylin-eosin (HE)) shows bland fibroblastic tumor cells and homogeneous hypocellular tissue, consistent with fibroedematous, hypovascular stroma. (b) FOSL1 immunohistochemistry.

**Figure 4:**
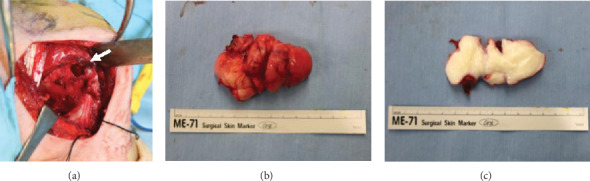
(a) Intraoperative photograph. A cavity was present in the lesser tubercle of the humerus. (b, c) Macroscopic appearance (b) and cut-surface view (c) of the tumor.

A 66 year-old woman experienced left shoulder pain without any history of trauma. She was diagnosed with periarthritis of the shoulder by a nearby clinic. Two months later, she noticed a small axillary lump that reached 1 cm in size. Due to her persistent shoulder pain and progressively enlarging axillary lump, she visited another hospital and was finally referred to our hospital for further evaluation and treatment. By that time, it had been 4 months since the onset of shoulder pain and 2 months since she became aware of the mass. Her physical examination revealed a hard, tender, palpable 6 × 5 cm mass with poor mobility under the left axilla and restricted movement of the left shoulder, especially external rotation (Figures 1(a) and 1(b)). Plain X-rays showed a well-circumscribed, oval, osteolytic lesion with sclerotic margins in the lesser tubercle of the left humerus (Figures 1(c) and 1(d)). Contrast-enhanced computed tomography (CT) showed a soft tissue mass with invasion of the adjacent left humerus (Figure 1(e)). On magnetic resonance imaging (MRI), the lesion measured 70 × 35 × 30 mm and appeared as a tumor with clear margins in the subscapularis muscles. The lesion had low signal intensity on T1- and T2-weighted images and short TI inversion recovery (STIR) (Figure 2). Mild internal enhancement was observed on gadolinium-enhanced fat-suppressed T1-weighted images (Figure 2). Because the tumor was a deep-seated lesion larger than 5 cm, there was a high likelihood that it was a sarcoma. A core needle biopsy was performed. Histological examination revealed a uniformly paucicellular tumor consisting of widely spaced, bland, spindle- to stellate-shaped cells embedded in a myxocollagenous stroma (Figure 3(a)). Immunohistochemical analysis showed that the tumor cells were diffusely positive for FOSL1 (Figure 3(b)) and were negative for *α*-smooth muscle actin, desmin, CD34, *β*-catenin, and S-100 (data not shown). The tumor was finally diagnosed as DFB and was marginally resected using a deltopectoral approach. The tumor was located in the patient's subscapularis muscle and invaded into the lesser tubercle of the left humerus, where a small cavity was observed after resection (Figure 4(a)). Macroscopically, the tumor was a well-circumscribed, firm, ovoid mass, while the cut surface was a homogeneously white-colored, solid mass (Figures 4(b) and 4(c)). Postoperatively, there was a significant improvement in pain and ROM of the patient's shoulder. MRI examination performed 1 year after the operation showed no recurrence, and there also was no evidence of recurrence at 18 months after the operation.

## 3. Discussion

DFB, also known as collagenous fibroma, is a benign, fibrous, soft tissue tumor first described by Evans in 1995 [1]. The condition occurs across a wide age range, with a median of 50 years, and exhibits male predominance [2]. The tumor shows a wide anatomic distribution but occurs most frequently in the upper body, including the arms, shoulders, and neck [2]. The diameter ranges from 1 cm to 20 cm (median, 3.0 cm) [2]. It has been reported that patients generally present with a history of a painless, slowly growing mass, often of relatively long duration.

Our report is unique from other published cases for several reasons. First, the clinical course was rather short, while Miettinen and Fetsch reported that tumors had been apparent for over 6 months in 57% of patients, and for over a year in 32% [2]. Second, our patient presented with pain and limited ROM of the shoulder, while the majority of patients with DFB are asymptomatic. Third, bone invasion is rare in DFB. A literature review showed reports of only 3 patients with DFB who presented with apparent bone invasion [3–5]. Aggressive clinical features, such as large tumor size or pain, were present in all patients, including the present case. Radiographically, the MRI findings of our patient were not much different from those in reported cases. Low signal intensity on T1- and T2-weighted images suggests the presence of collagen-rich, fibrous tumors. Yamamoto et al. reported that peripheral rim enhancement with gadolinium could be diagnostic in discriminating DFB from other fibrous tumors [6]. However, histological examination is necessary for a definitive diagnosis.

For the differential diagnosis in this case, fibrous soft tissue lesions with bone invasion were considered, particularly desmoid-type fibromatosis (desmoids). Morphologically, there are some differences between desmoids and DFB. Desmoids are composed of uniform spindle-shaped cells and often form poorly defined fascicles within a collagenous stroma. The degree of cellularity is generally moderate. Desmoids infiltrate into the surrounding tissue, which may result in a high risk of local recurrence, even after margin-free resection. On the other hand, DFB is characterized by a paucicellular lesion with spindle- and stellate-shaped fibroblastic cells embedded in a hypovascular, densely fibrous stroma, which is why this lesion is also known as collagenous fibroma. Nevertheless, desmoid would have some resemblance to DFB. Indeed, in an early case series of DFB, desmoids were the most frequent initial pathological diagnosis registered in Soft Tissue Registry of the Armed Forces Institute of Pathology between 1970 and 1995 [2]. Therefore, a diagnostic marker discriminating between DFB and other fibrous tumors including desmoids is needed. Recently, cytogenetic analyses revealed chromosomal rearrangement involving chromosome 11, which is proposed to result in an aberrant expression of FOSL1 [7, 8]. Kato et al. reported that FOSL1 may be a diagnostic aid for differentiating DFB from other histological mimics [9]. In the present case, FOSL1 was expressed in tumor cell nuclei, while there was no expression of *β*-catenin.

A previous study reported that DFB lesions were predominantly subcutaneous, that one-quarter of cases involved the skeletal muscle, and that microscopically, most tumors infiltrated into subcutaneous fat and 27% extended into the skeletal muscle [2]. Therefore, DFB may basically have an invasive potential. In addition, her short clinical course and large tumor size suggest that DFB in our patient would be more aggressive than DFB in previous reports [2, 10]. Furthermore, in our case, because macroscopic observation revealed that DFB purely occurred in the subscapularis muscle. Therefore, we speculate that such invasive potential, aggressiveness, pure intramuscular involvement, and anatomical site dependency (i.e., subscapularis muscle) may have contributed to the invasion into the lesser tubercle of the humerus through the muscle's insertion site, thus causing the formation of the humeral cavity. It is still unclear whether the tumor itself directly caused pain. We speculate instead that tumor invasion from the subscapularis muscle into the humerus may have caused both pain with motion and limited ROM of the shoulder.

While wide local excision with negative pathologic margins is the treatment of choice for most desmoids, simple excision is indicated for DFB. The prognosis of DFB is good despite its potential invasiveness. No local recurrence has been reported in any previous cases, including the 3 abovementioned patients with bone invasion [3–5].

## 4. Conclusion

The present article reports a rare case of shoulder DFB with bone invasion. Preoperative biopsy is necessary to distinguish DFB from other large fibrous tumors with bone invasion, and FOSL1 may be helpful for the diagnosis of DFB. Even if DFB with bone invasion is symptomatic, accurate preoperative diagnosis can lead to simple excision.
